# Invisibility as a structural determinant: Mortality outcomes of Asians and Pacific Islanders experiencing homelessness

**DOI:** 10.3389/fpubh.2022.969288

**Published:** 2023-01-06

**Authors:** Jamie Suki Chang, Katherine Saxton, Georgia Bright, Maya S. Ryan, E. Francis Lai, Michelle A. Jorden, Andy Gutierrez

**Affiliations:** ^1^Department of Public Health, Santa Clara University, Santa Clara, CA, United States; ^2^Office of the Medical Examiner-Coroner, San Jose, CA, United States; ^3^Office of the Public Defender Post-Conviction Outreach Unit, San Jose, CA, United States

**Keywords:** homeless, cause of death, mortality, Asian, API, structural determinants, exclusion, isolation

## Abstract

**Introduction:**

Asians and Pacific Islanders (APIs) who are experiencing homelessness are situated in a social intersection that has rendered them unrecognized and therefore vulnerable. There has been increasing attention to racial disparities in homelessness, but research into API homelessness is exceedingly rare, despite rapidly growing populations. The purpose of this study is to examine the causes of death among APIs who died while homeless in Santa Clara County (SCC) and compare these causes to other racial groups.

**Materials and methods:**

We report on data obtained from the SCC Medical Examiner-Coroner's Office on unhoused people's deaths that occurred between 2011 and 2021 (*n* = 1,394), including data on deaths of APIs experiencing homelessness (*n* = 87).

**Results:**

APIs comprised 6.2% of total deaths of unhoused people. APIs died less often of causes related to drug/alcohol use than all other racial groups (24.1, compared to 39.3%), and there was a trend toward more API deaths from injuries or illnesses. When APIs were disaggregated into sub-groups (East/Southeast Asian, South Asian, Pacific Islander), there were notable mortality differences in cause of death, age, and sex.

**Discussion:**

We argue that invisibility is a structural determinant of health that homeless APIs face. Though relatively small in numbers, APIs who are invisible may experience increased social isolation and, subsequently, specific increased mortality risks. To understand the health outcomes of unhoused APIs, it is essential that researchers and policymakers recognize API homelessness and gather and report disaggregated races and ethnicities.

## Introduction

Asians and Pacific Islanders (APIs) experiencing homelessness are situated in a social intersection that has rendered them invisible and unacknowledged in public discourse and social policies. In recent years, major public health organizations (1, 2) have declared that homelessness (i.e., being homeless, unhoused, houseless) is a public health crisis that is known to have profound, lasting health impacts on the individual and community (3). There is growing attention to racial health disparities in unhoused populations, but research and reporting into API homelessness remains exceedingly rare.

The sparse attention to unhoused APIs is due in part to the relatively small numbers. According to the 2019 Annual Homeless Assessment Report to Congress, APIs comprise just 2.9% of the national unhoused population (Asian 1.3% and Pacific Islander 1.6%), compared to 47.7% White, 39.8% Black, 22% Latino, 6.5% Multiple Races, and 3.2% Native American (4). Such statistics give the impression that homelessness is not a serious concern for APIs. However, the percentage of unhoused people who are API is higher than national averages in regions with large API populations. According to the 2022 Point-in-Time-Count (PITC), a bi-annual estimate of homeless counts and demographics, APIs make up 12% of the homeless population in San Francisco, and 9% in San Jose (5, 6). These numbers are increasing. Nationally, there has been record growth in APIs experiencing homelessness, with rates of Asian unsheltered homeless (7) and API sheltered homeless groups (8) growing faster in recent years, compared to other racial groups. In San Jose, California, between 2017–2020, the estimated population of unhoused APIs increased 57% (7, 9).

It is noteworthy that estimates of unhoused APIs are likely undercounts. Estimates of unhoused people rely on contacts with service providers, but APIs are less likely to access homeless services. Nationally, unhoused API people are less likely to stay in shelters compared to other racial groups, with the exception of Native Americans (9). One study of homeless veterans found that API veterans were more likely to screen positive for housing instability following discharge than their White counterparts; however, APIs were the racial group least likely to receive housing services (10). Advocates have argued that the PITC is susceptible to undercounting APIs due to systemic barriers like language differences or concern about disclosing immigration and citizenship status. Studies also indicate that unhoused and precariously housed APIs are more likely to stay in “doubled-up” settings (11, 12)—that is, in a home without being a member of the household—which is not captured by the PITC.

There is a serious dearth of public health research on API homelessness, but the existing studies do show that APIs have different patterns of risk factors and vulnerabilities in homelessness than other racial groups. One study found that for Asians, homelessness risk factors included having mood disorders, receiving welfare services, and having physical health conditions (13). Another study of homeless veterans showed that unhoused APIs were less likely to report having alcohol or drug issues than other racial groups, but they were significantly more likely to report current mental health issues and histories of psychiatric hospitalization (14). In a randomized control trial of Hepatitis A and B vaccine treatment among homeless parolees, being an unhoused API was one of the strongest predictors of non-completion of the vaccine (15).

Mortality data are an epidemiological surveillance tool that can provide insights into the health of unhoused people and the heightened risks they face. In the last 20 years, studies have shown that veterans, older adults, youths aged 15–25, and children under 18 who are unhoused are at greater risk of mortality than their housed counterparts (16–19). The primary causes of death vary by region, and they often include deaths related to substance use, injuries, and illnesses including cancer and heart disease (20–22).

Although most recent academic studies on homeless mortality report and analyze race, many do not include APIs (22–24). In studies that do include APIs, the sample sizes of APIs are too small for analysis (19, 25). To our knowledge, no prior academic study has analyzed unhoused API mortality.

The purpose of this study is to fill this gap by examining and reporting the causes of death among APIs who died while homeless in Santa Clara County (SCC), a region better known as Silicon Valley. SCC has a large population of Asian residents −39% Asian and 0.5% Pacific Islander (26). In examining mortality data, our intention is to characterize unhoused API deaths, which, given the absence of other data, can provide rare insights into the circumstances APIs face when homeless.

## Methods

We report on data obtained from the SCC Office of the Medical Examiner-Coroner (OMEC) on the deaths of people experiencing homelessness in SCC between 2011 and 2021 (*n* = 1,394). The OMEC determined a deceased individual was unhoused based on a medicolegal death investigation that included examining the circumstances and environment attending a death, interviewing people who knew the deceased individual, and verifying with next-of-kin. The data included: race/ethnicity, age, sex, location of death, cause of death, manner of death, and other notable conditions.

### Cause of death categories

The causes of death were recorded as clinical descriptions on the death certificate, rather than International Statistical Classification of Diseases (ICD) codes. We classified these causes of death into six mutually exclusive categories. We first categorized Homicides, Suicides, and Undetermined cases; these determinations were made by the OMEC. Then we identified cases involving Substance Use if one or more drugs and/or alcohol were mentioned in the primary cause of death. Finally, we categorized the remaining cases as either Injury or Illness.

### Race/ethnicity categories

The race/ethnicity of the deceased individual was included in the OMEC data. Racial and ethnic categories included: Alaskan Native, American Indian, Asian, Black, Hispanic, Native Hawaiian, Pacific Islander, South Asian, Unknown, and White. We combined the OMEC's Asian, South Asian, and Pacific Islander categories to create our API category. We also disaggregated the API category into three sub-groups: East/Southeast Asians, South Asians, and Pacific Islanders.

### Statistical analysis

We analyzed descriptive statistics of homeless decedents in SCC from 2011 to 2021, comparing age and cause of death among API decedents to all other racial groups combined. Descriptive statistics included counts, means, percentages, and confidence intervals. All analyses were performed using Stata 15 (College Station, TX) and Microsoft Excel.

## Results

In SCC, between 2011 and 2021, there were 87 unhoused API deaths, which made up 6.2% of total deaths of unhoused people in the county (Tables 1, 2). The sex distribution of deaths was consistent between API and non-API racial groups. Men made up 83.9% of API deaths (95% CI: 74.5–90.9%) and 81.6% of non-API deaths (95% CI: 79.4–83.6%).

**Table 1:** Characteristics of the sample (*n* = 1,394), including APIs (*n* = 87) and sub-groups.

**Characteristic**	**Total (*n* = 1,394)**
**Race/ethnicity** API East/SE Asian South Asian Pacific Islander Hispanic Black White Native American Middle Eastern Other Unknown	***n*** **(%)** 87 (6.2%) 70 (5.0%) 4 (0.3%) 13 (0.9%) 438 (31.4%) 132 (9.5%) 696 (49.9%) 14 (1.0%) 7 (0.5%) 12 (0.9%) 8 (0.6%)
**Sex** Male Female Unknown	***n*** **(%)** 1,139 (81.7%) 243 (17.4%) 12 (0.9%)
**Age** Younger than 18 18–25 26–30 31–40 41–50 51–60 61–70 71–80 81+ Unknown	***n*** **(%)** 8 (0.6%) 36 (2.8%) 48 (3.4%) 166 (11.9%) 290 (20.8%) 432 (31.0%) 308 (22%) 75 (5.4%) 14 (1.0) 17 (1.2%)

**Table 2:** Characteristics of the API sub-groups (*n* = 87).

**Characteristic**	**East/SE Asian (*n* = 70)**	**South Asian (*n* = 4)**	**Pacific Islander (*n* = 13)**	**Total (*n* = 87)**
**Sex** Male Female Unknown	***n*** **(%)** 60 (85.71%) 9 (12.86%) 1 (1.43%)	***n*** **(%)** 3 (75.00%) 1 (25.00%) 0 (0.00%)	***n*** **(%)** 10 (76.92%) 3 (23.08%) 0 (0.00%)	***n*** **(%)** 73 (83.91%) 13 (14.94%) 1 (1.15%)
**Age** Younger than 18 18–25 26–30 31–40 41–50 51–60 61–70 71–80 81+ Unknown	***n*** **(%**) 0 (0.00%) 1 (1.43%) 0 (0.00%) 7 (10.00%) 13 (18.57%) 20 (28.57%) 18 (25.71%) 7 (10.00%) 2 (2.86%) 2 (2.86%)	***n*** **(%)** 0 (0.00%) 0 (0.00%) 0 (0.00%) 0 (0.00%) 2 (50.00%) 1 (25.00%) 1 (25.00%) 0 (0.00%) 0 (0.00%) 0 (0.00%)	***n*** **(%)** 0 (0.00%) 1 (7.69%) 3 (23.08%) 3 (23.08%) 2 (15.38%) 3 (23.08%) 1 (7.69%) 0 (0.00%) 0 (0.00%) 0 (0.00%)	***n*** **(%)** 0 (0.00%) 2 (2.30%) 3 (3.45%) 10 (11.49%) 17 (19.54%) 24 (27.59%) 20 (22.99%) 7 (8.05%) 2 (2.30%) 2 (2.30%)

### Cause of death among APIs

**Figure 1:**
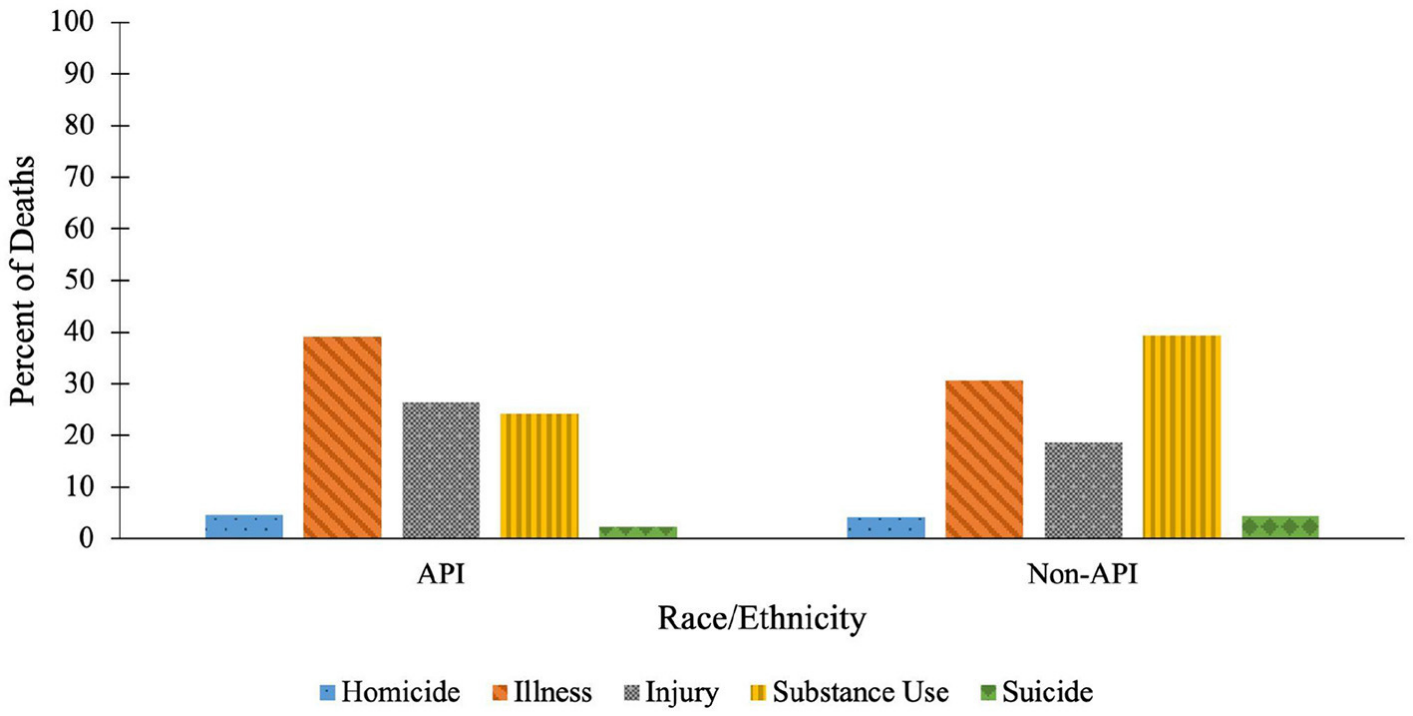
Distribution of causes of death among APIs vs. all other racial groups. APIs (*n* = 87) are compared to all non-API racial groups combined (*n* = 1,307).

The distribution of causes of death differed between APIs and all other racial groups combined (Figure 1). APIs were less likely to die of causes related to substance use (24.1%, 95% CI: 15.6–34.5%) than all other racial groups combined (39.3%, 95% CI: 36.7–42.0%). Unhoused APIs were more likely to die of illness (39.1%, 95% CI: 28.8–50.1%) than other racial groups (30.7%, 95% CI: 28.1–33.3%) and more likely to die from injuries (26.4%, 95% CI: 17.6–37.0%) than other racial groups (18.5%, 95% CI: 16.5–20.8%), although these differences were not statistically significant.

Illness deaths among APIs were most commonly cardiovascular disease (50.0%, 95% CI: 32.4–67.6%) and infection (11.8%, 95% CI: 3.3–27.5%). The majority of injury deaths were due to blunt force injury (73.9%, 95% CI: 51.6–89.8%). Most deaths attributed to substance use involved acute toxicity from stimulants, such as methamphetamine (61.9%, 95% CI: 38.4–81.9%) and cocaine (14.3%, 95% CI: 3.0–36.3%).

### Age of death among APIs

The age of death of unhoused APIs (54.0 years, 95% CI: 51.0–56.9%) was consistent with the overall average age of death for all racial groups (52.5 years, 95% CI: 51.0–56.9%). However, API women (57.9 years, *n* = 13, 95% CI: 46.8–69.1%) showed a wider range in age of death, compared to API men (53.3 years, *n* = 72, 95% CI: 50.3–56.2%). There were two deaths among API women over 85 years of age (85 and 87 years), whereas the oldest death among API men was 77 years.

### Cause of death by API sub-groups

Due to small sample sizes, differences in causes of death between API sub-groups are not statistically significant. However, the distribution of causes of death differed between disaggregated API sub-groups of East/Southeast Asian, Pacific Islander, and South Asian decedents. Among East/Southeast Asian unhoused deaths (*n* = 70), 45.7% were due to illness (95% CI: 33.7–58.1%), 28.5% to injury (95% CI: 18.4–40.6%), and 18.6% to substance use (95% CI: 10.3–29.7%). For Pacific Islanders (*n* = 13), 30.7% of deaths were due to substance use (95% CI: 9.1–61.4%), 23.1% to homicide (95% CI: 5.0–53.8%), 23.1% to injury (95% CI: 5.0–53.8%), and only 15.4% to illness (95% CI: 1.9–45.4%). All South Asian unhoused deaths (*n* = 4) were related to substance use.

### Age of death by API sub-groups

Age of death also differed between disaggregated API sub-group categories. The average age of death for East/Southeast Asian unhoused people was 56.8 (*n* = 68, 95% CI: 53.7–59.8%) years, for South Asian individuals was 51.0 (*n* = 4, 95% CI: 38.2–63.8%), and age of death was notably lower at 40.5 years for Pacific Islanders (*n* = 13, 95% CI: 32.6–48.3%). East/Southeast Asian women had the oldest age at death among APIs, with an average age of 65.7 years old (*n* = 9, 95% CI: 54.1–77.2%), which was over 10 years older than the average age of death for East/Southeast Asian men at 55.4 years (*n* = 59, 95% CI: 52.4–58.4%).

## Discussion

To summarize, we found that deaths of unhoused APIs in SCC were attributed to distinct causes compared to other racial groups, with notable gender and age differences. APIs were more likely to die from causes related to illness and injury and less likely to die of causes related to substance use, compared to other racial groups. We also show that when the API category is disaggregated into sub-groupings, there were age, sex, and cause of death differences between unhoused East/Southeast Asian, South Asian, and Pacific Islander people.

There are several limitations to this study. First, the sample size per year of unhoused people's deaths, while tragically high, is small for statistical analysis. We describe unhoused people's deaths in SCC, but this analysis is not necessarily predictive or generalizable to other regions. The OMEC provided the causes of death as clinical descriptions. We interpreted the clinical descriptions to standardize into the cause of death categories used for analysis. The OMEC data did not include separate categories for East Asian and Southeast Asian decedents. This study involved people who died while unhoused, whose cases were reviewed by the OMEC, and does not include cases that were not reviewed through this office. The findings describe mortality trends, but do not determine the scale of death that is directly attributable to being unhoused. Factors such as immigration status, shelter status, and duration of homelessness are key factors that we were unable to include in this analysis.

These limitations notwithstanding, there are several implications for research and policy surrounding API homelessness. First, in recent conversations addressing anti-Asian hate, API advocates (27–29) and health researchers (30) have brought attention to API invisibility—the failure to meaningfully represent API experiences and needs. They argue that invisibility lack of recognition, exclusion, is a form of racism that APIs face, resulting in othering, dehumanization, and violence.

We argue that the failure to represent unhoused APIs in health research, advocacy, media, and policy is an upstream structural determinant that renders them more vulnerable to specific risks (31). In the words of Kimberlie Crenshaw (32), “When you can't see a problem, you can't solve it.” We hypothesize that API invisibility leads to lack of community outreach and social support and consequently, heightened isolation. Isolation while homeless has been linked to serious health consequences, including increased depression, self harm, and exposure to violence (33, 34). Consistent with this framework, in our study we found that APIs, who are seldom the focus of health and homelessness research or interventions, die of tragic and preventable causes, with high rates of injury-related causes of death (e.g. blunt force injury).

In many regions, APIs face homelessness with little to no targeted outreach and support. Public health programming, interventions, education, and prevention that are tailored to APIs based on data are necessary to prevent the injurious, tragic outcomes we report in this analysis.

The information drawn from mortality research can be used to develop public health interventions and research investigations focused on unhoused APIs. For example, we found that APIs were more likely to die of injury deaths than other racial groups, and that these injuries were most commonly blunt force injuries. This outcome should be investigated further to understand the causes and contexts of these blunt force injury deaths. We also found high rates of substance use deaths among South Asians, early mortality among Pacific Islanders, and substantially older deaths among homeless East/Southeast Asian women. Although the sample sizes were small for these groups, recognizing these patterns can lead to targeted health interventions, such as developing methamphetamine outreach for unhoused South Asians and investigating the pathways to homelessness for Pacific Islanders and older East/Southeast Asian women.

For decades, API researchers have advocated for the disaggregation of API data by ethnicity or at least by API sub-groups (e.g. East Asians, South Asian, Southeast Asian, Pacific Islander) (35, 36). Our findings, which suggest differences between API sub-groups, are further evidence of the need for disaggregation. Key differences in mortality—for example, that unhoused Pacific Islanders died over 15 years younger than East Asians—are blurred and effectively erased without disaggregated data. These findings are consistent with a large body of health research showing major health differences and disparities between API ethnicities (37, 38). We add to the chorus of researchers urging for the use of survey instruments with disaggregated API categories and the reporting on disaggregated API data categories when possible.

Finally, beyond the implications for unhoused APIs, this analysis underscores the need to improve the ways health researchers recognize minority and underrepresented groups in homeless research. APIs are not the only group who are misrepresented when data are broadly aggregated. When the largest racial groups (or dominant groups of any social category, including genders, sexualities, ages, religions, etc.) are solely reported, groups that fall outside the largest demographics are oversimplified, obscured, and even erased, potentially furthering their marginalization.

Today, APIs face increased homelessness (7–9) as well as increasing housing insecurity (39). Unhoused people from all racial backgrounds need much greater support, but our findings reveal that APIs, though relatively small in numbers, may face specific heightened risks while unhoused. By examining the unique circumstances faced by homeless APIs, research like this can address the structural vulnerability of invisibility, which is the first step toward developing tailored public health interventions to prevent tragic health outcomes.

## Data availability statement

The original contributions for this study are based on publicly available data through the Santa Clara County Office of the Medical Examiner-Coroner. This open data portal (https://medicalexaminer.sccgov.org/medical-examiner-coroner-dashboard) compiles and summarizes data from unhoused people's autopsy reports, including year of death, age, sex, and the primary cause of death. Further inquiries can be directed to the corresponding author/s.

## Ethics statement

This study, which involved deceased human subjects, was reviewed and approved by the Institutional Review Board at Santa Clara University Office of Research Compliance. The data are publicly available, and written informed consent for participation was not required for this study in accordance with national legislation and the institutional requirements.

## Author contributions

JC and KS were the supervisors of the study, involved in manuscript development, and writing. MJ and AG were involved in the original gathering of cause of death data and consulted on analysis. GB, EL, MR, and KS were involved in the analysis of the project, including data management, categorizing and coding data, statistical analysis, developing figures, and tables. All authors contributed to the article and approved the submitted version.
